# Long-read sequencing reveals chromothripsis in a molecularly unsolved case of Cornelia de Lange syndrome

**DOI:** 10.3389/fgene.2024.1358334

**Published:** 2024-03-13

**Authors:** Ilaria Bestetti, Milena Crippa, Alessandra Sironi, Matteo Bellini, Francesca Tumiatti, Sara Ballabio, Ferruccio Ceriotti, Luigi Memo, Maria Iascone, Lidia Larizza, Palma Finelli

**Affiliations:** ^1^ SC Patologia Clinica, SS Laboratorio Genetica Medica, Fondazione IRCCS Ca’ Granda Ospedale Maggiore Policlinico, Milano, Italy; ^2^ Laboratorio Sperimentale di Ricerche di Citogenetica Medica e Genetica Molecolare, IRCCS Istituto Auxologico Italiano, Milano, Italy; ^3^ Laboratorio di Genetica Medica, ASST Papa Giovanni XXIII, Bergamo, Italy; ^4^ SC Genetica Medica, IRCCS Burlo Garofolo, Trieste, Italy; ^5^ Dipartimento di Biotecnologie Mediche e Medicina Traslazionale, Università degli Studi di Milano, Milano, Italy

**Keywords:** long-read sequencing, NIPBL, Cornelia de Lange syndrome, translocation, complex chromosomal rearrangement, chromothripsis, RT-qPCR, bkps mapping

## Abstract

Thanks to a long-read sequencing (LRS) approach, in this study, we have reported a molecularly solved case of a proband with a clinical diagnosis of Cornelia de Lange syndrome (CDLS), which is a multisystemic disorder whose causative molecular defects involve cohesin complex genes, with *NIPBL* located at 5p13.2 accounting for approximately 50%–60% of CDLS cases. The first-tier tests revealed an abnormal karyotype 46,XY,t(5;15)(p13;q25)dn and a preserved *NIPBL* sequencing. Copy number variants (CNVs) at the translocation breakpoints, in disease genes, or in probably pathogenic loci were excluded by a-CGH analysis. Through fluorescence *in situ* hybridization (FISH) analysis on derivative chromosome 5, the breakpoint was relocated 3 Mb far from *NIPBL* 5′UTR, which seemed fully maintained as FISH-probe mapping to the gene showed no split signals. Moreover, tri-color FISH revealed an apparently balanced paracentric inversion including *NIPBL* on derivative 5. Based on the strong clinical suspicion, we evaluated the *NIPBL* transcript by RT-qPCR that revealed a normal amount of transcript till exon 22 and a halved amount of the transcript from exon 23 to 3′UTR, indicating the expression of a truncated transcript probably leading to a defective protein. Despite RT-qPCR confirmed the patient’s CDLS clinical diagnosis, the molecular mechanism underlying this event remained to be an unsolved challenge for years. The LRS approach with nanopore technologies was able to fill the gap in this complex scenario and highlighted a chromothripsis event marked out at 5p13.2 by 36 breaks clustered in a 7.3-Mb region. The *NIPBL* gene was disrupted by 16 breaks and the resulting fragments were relocated in different positions and orientations. LRS confirmed the previous findings, and it has been proven to be crucial to define the complex chromosomal rearrangement in this patient which escaped current diagnostic investigations. Its application in the clinical practice will contribute to solve the unsolved.

## 1 Introduction

Cornelia de Lange syndrome (CDLS; OMIM #122470, #300590, #300882, #610759, and #614701) is a rare multisystem disorder affecting 1:10,000 and 1:30,000 live births (Kline et al., 2007; Mannini et al., 2013), which is characterized by distinctive facial features, growth retardation, intellectual disability, upper limb malformations, hirsutism, and other abnormalities’ variable expressivity (Jackson et al., 1993). CDLS is not only a clinically heterogeneous disorder but also shows genetic heterogeneity. To date, five genes (i.e., *NIPBL*, *SMC1A*, *SMC3*, *RAD21*, and *HDAC8*) have been associated with the CDLS classic phenotype (Krantz et al., 2004; Tonkin et al., 2004; Musio et al., 2006; Deardorff et al., 2007; Deardorff et al., 2012; Deardorff et al., 2012; Yuan et al., 2019), and *NIPBL* is considered the major gene as it accounts for 70%–80% of the loss-of-function defects in the patients (Kaiser et al., 2014; Boyle et al., 2015). Two other genes, *BRD4* which encodes a protein interacting with *NIPBL* (Olley et al., 2018) and *ANKRD11* (Parenti et al., 2016), have been implicated in non-classic CDLS phenotype, and variants in genes, functionally linked to cohesin, such as *AFF4* (Izumi et al., 2015), have been identified in individuals sharing limited signs of CDLS (Kline et al., 2018). Due to the wide and undefined phenotypic spectrum, only 70% of individuals with a CDLS clinical diagnosis are confirmed by genetic tests, suggesting the existence of additional yet unidentified loci or unsolved disease mechanisms. Indeed, in approximately 5% of cases negative to the standard mutation flowchart, various chromosome rearrangements affecting *NIPBL* have been reported, including rare balanced chromosome translocations at 5p13.2 (Krantz et al., 2004; Tonkin et al., 2004; Hayashi et al., 2007), complete gene deletions, and intragenic imbalances (Bhuiyan et al., 2007; Ratajska et al., 2010; Pehlivan et al., 2012; Russo et al., 2012; Kuzniacka et al., 2013; Cheng et al., 2014). Recently, a complex chromosomal rearrangement (CCR) solved by whole-genome sequencing (WGS) has been described in an infant with a clinical diagnosis of CDLS (Plesser Duvdevani et al., 2020). Here, we report a molecularly unsolved case of a proband with a clinical diagnosis of CDLS1, whose pathomechanism could be unveiled using long-read sequencing (LRS). LRS was crucial to reconstruct the origin and composite structure of the patient’s CCR which escaped current diagnostic investigations.

## 2 Case presentation and methods

### 2.1 Clinical report

The proband, actually an 18-year-old boy, was born to non-consanguineous healthy parents at the 37th week of gestation by cesarean section due to severe intrauterine growth retardation. His birth weight was 1,920 g (<3rd centile), length was 40 cm (<3rd centile), and head circumference was 29.8 cm (<3rd centile) (Apgar: 1′ = 6; 5′ = 7; 10′ = 10). The proband’s parents had a total of five pregnancies, with the first resulting in the birth of the present case and the remaining four completed pregnancies resulting in the birth of three females and one male, all in good health. In all four pregnancies following the birth of the proband, amniocentesis had been performed, which showed no abnormalities. The patient’s clinical (gestalt) diagnosis was made at birth, where he revealed craniofacial features typical of CDLS1 (microcephaly, synophrys, highly arched eyebrows, long eyelashes, short nasal bridge with anteverted nares, and cleft palate) and also hypertrichosis, heart abnormalities (intraventricular defects, patent foramen ovale, and stenosis of the left pulmonary branch), pyloric stenosis, cryptorchidism, hypospadias, and small hands. During the follow-up, the patient underwent several surgeries: at ∼1 month of age, he underwent extramucosal pyloromyotomy surgery for pyloric stenosis; at 8 months, right orchidopexy was performed; at 18 months, left orchidopexy was performed; at 1 year, plastic soft palate was fixed; and at 2 years, plastic hard palate was fixed. Moderate sensorineural hearing loss was definitively established at 2 and a half years with subsequent prosthesization; at 4 years, very severe kyphosis of the spine began to be expressed, and surgery for the right cataracts was made when he was 14 years old. Moreover, regarding stature-ponderal development, the patient showed persistence in microcephaly, height, and weight below the fifth centile also due to severe gastroesophageal reflux and recurrent respiratory infections. As for neuromotor development, the patient presented a very severe developmental delay. He pronounced the first bisyllables only after the age of 7 years and he never walked independently.

### 2.2 Molecular screening and MLPA analysis

Genomic DNA was extracted from whole blood using the GenElute Blood Genomic DNA kit (Sigma-Aldrich, St. Louis, MO) and molecular screening was performed to search for pathogenic variants of *NIPBL* (NM_133433) using denaturing high-performance liquid chromatography (DHPLC) with intronic exon flanking primers along the whole-coding sequence, followed by Sanger sequencing on an ABI PRISM 3130 sequencer (Applied Biosystems, Foster City, CA) as described previously (Selicorni et al., 2007). Pathogenic variants of the *SMC1A* (NM_006306) gene were screened by Sanger sequencing using intronic exon flanking primers (Musio et al., 2006). Electropherograms were analyzed with ChromasPro software 1.42 (Technelysium Pty Ltd., Tewantin QLD, Australia). The SALSA P141/P142 *NIPBL* MLPA kit (MRC-Holland, Amsterdam, Netherlands) was used in accordance with the manufacturer’s instructions. MRC-Coffalyser v9.4 software was used for data interpretation.

### 2.3 Cytogenetic analysis

Conventional cytogenetic analysis was performed on 50 QFQ-banded metaphases obtained from the proband’s and parents’ peripheral blood lymphocytes using standard procedures. The karyotypes were described in accordance with ISCN (2016) (ISCN, 2016, 2016).

### 2.4 Array-CGH analysis

High-resolution array-based comparative genomic hybridization (a-CGH) analysis was performed on genomic blood DNA of the patient, using the SurePrint G3 Human CGH microarray kit 1 × 1M in accordance with the manufacturer’s instructions (Agilent Technologies, Palo Alto, CA). Copy number variants (CNVs) were mapped using the human genome assembly GRCh38/hg38 and were considered rare if unreported or reported at a very low frequency (≤0.05%) according to the Database of Genomic Variants (DGVs) (http://projects.tcag.ca/variation/, released in March 2016). CNV classification by clinical relevance was performed according to the guidelines suggested by Miller et al. (2010) and successively by the American College of Medical Genetics (Kearney et al., 2011).

### 2.5 Fluorescence *in situ* hybridization analysis

FISH analyses on metaphases were performed using BAC and Fosmid clones as probe, selected using the UCSC Genome Browser (University of California Santa Cruz, reference genome assembly GRCh37/hg19), and provided by Invitrogen Ltd. (Carlsbad, CA) and the Children’s Hospital Oakland Research Institute (CHORI) (Oakland, CA). All clone DNAs were labeled by nick-translation with Cy3-dUTP (Amersham, Chalfont St. Giles, UK), and digoxigenin (Hoffman-La Roche, Basel, Switzerland), and then visualized with FITC-anti-digoxigenin antibodies (Hoffman-La Roche). FISH experiments were performed using standard procedures (Lichter and Cremer, 1992), and, on average, => 20 metaphases per each BAC clone were analyzed. Olympus BX61 fluorescence microscope and CytoVision 7.4 software were used for metaphase detection and image acquisition.

### 2.6 *NIPBL* expression analysis

The RNA of the patient and five healthy controls was collected and isolated using the Tempus Blood RNA tubes and the Tempus Spin RNA Isolation Kit (Thermo Fisher Scientific, Waltham, MA), and reverse-transcribed with the High-Capacity cDNA Reverse Transcription Kit (Thermo Fisher Scientific). RT-qPCR (reverse transcription quantitative PCR), based on TaqMan methodology, was performed using an ABI PRISM 7900HT Sequence Detection System (Applied Biosystems, Foster City, CA). The amounts of *NIPBL* mRNAs were calculated using the 2^−ΔΔCT^ method, with *GAPDH* and *TBP* as endogenous normalizing genes. All assays were provided by Thermo Fisher Scientific (TaqMan Gene Expression assays: *NIPBL* ID# Hs01122280_m1, exons 11–12; Hs01122252_m1, exons 20–21; Hs01122253_m1, exons 21–22; Hs01122254_m1, exons 22–23; Hs01122255_m1, exons 23–24; Hs01122256_m1, exons 24–25; Hs01122257_m1, exons 25–26; Hs01122259_m1, exons 27–28; Hs01122262_m1, exons 29–30; Hs01122269_m1, exons 36–37; *GAPDH* ID# Hs99999905_m1; *TBP* ID# Hs00427620_m1). RT-qPCR data were analyzed using the RQ Manager 1.2 software (Thermo Fisher Scientific). We established a range of normal gene expression using five healthy controls and calculating the mean value ±2 standard deviations (SDs). If the expression level in the patient was out of the control range, a dysregulation of the index gene could be inferred.

### 2.7 Long-read sequencing, PCR mapping, and validation of genomic breakpoints

Patient genomic DNA was fragmented using g-TUBE (Covaris) to obtain a final size of approximately 10 kb, and 1µg of gDNA was subjected to sequencing. DNA end-prep and adapter ligation were performed using the NEBNext companion module for Oxford Nanopore Technologies (ONT) Ligation kit (New England BioLabs). Sequencing library was prepared with the Ligation Sequencing Kit SQK-LSK110 (Oxford Nanopore Technologies) and run on a R9.4.1 Flowcell on the MinION Mk1B (Oxford Nanopore Technologies). DNA purification during library preparation was performed using Agencourt AMPure XP beads (Beckman Coulter). Sequencing was performed for 72 h and base-calling was performed using guppy_basecaller (v.6.5.7). An alignment to the reference genome GRCh38 was carried out using minimap2 (v.2.17), and sniffles (v.1.0.11) was applied for structural variant calling. The final structure and breakpoint coordinates were deduced by manual inspection using the Integrative Genomics Viewer (IGV) (v.11.0.13). Each of the identified breakpoints was validated by PCR amplification and Sanger sequencing. PCR reactions were performed using the Phire Green Hot Start II DNA Polymerase kit (Thermo Fisher Scientific), and the amplicons were purified and sequenced using the BigDye^®^ Terminator v3.1 Cycle Sequencing Kit (Thermo Fisher Scientific). Electropherograms were analyzed using ChromasPro 1.5 software (Technelysium Pty Ltd., Tewantin, QLD, Australia). Primers used for validation are listed in Supplementary Table S1.

## 3 Results

### 3.1 Molecular screening and MLPA analysis

The mutational screening of *NIPBL* and *SMC1A* genes revealed wild-type sequences and the MLPA analysis excluded the occurrence of whole-exon deletions/duplications in *NIPBL*.

### 3.2 Cytogenetic analysis

Conventional cytogenetic analysis on QFQ-banded metaphase chromosomes prepared from peripheral blood lymphocytes showed in the proband a *de novo* apparently balancing reciprocal chromosome translocation between the short arm of chromosome 5 and the long arm of chromosome 15 [46,XY,t(5;15)(p13;q25)] (Supplementary Figure S1).

### 3.3 Array-CGH analysis

The high-resolution 1 x 1M array-based comparative genomic hybridization analysis (a-CGH, Agilent Technologies) ruled out microdeletion/duplications spanning the translocation breakpoints (bkps) chromosomal bands supporting the view of an apparently balanced rearrangement. The rare CNVs identified (Table 1) do not encompass genes related to the CDLS phenotype and were classified as likely benign according to the ACMG guidelines.

**TABLE 1:** Copy number variants identified by a-CGH.

chr	Start^a^	End^a^	bp	CN	Inheritance	Clinical classification
7p22.1	6297469	6323285	25,817	1	Maternal	Likely benign
13q12.3	30697928	30911752	213,825	3	Maternal	Likely benign
17q11.2	29691281	29726392	35,112	3	Paternal	Likely benign

^a^ hg38 genomic coordinates.

CN, copy number.

### 3.4 FISH analyses

**FIGURE 1:**
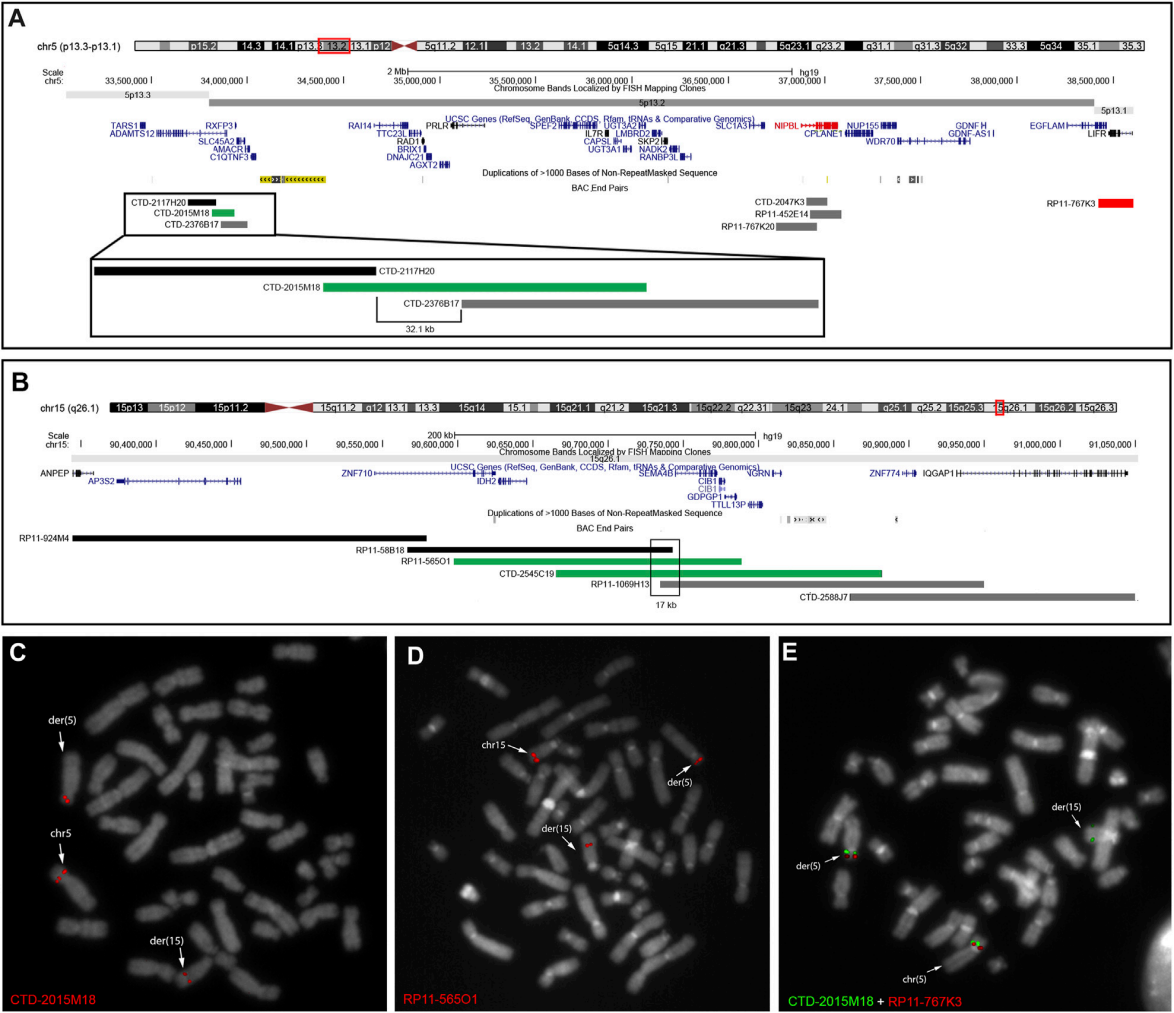
FISH analyses used to characterize patient’s rearrangement. **(A)** UCSC Genome Browser showing the 4-Mb region involved in the translocation and chromosomal inversion on der(5) at 5p13.3–p13.1. BAC probes are colored in black when mapping on der(15) and in gray when mapping on der(5). BAC probe CTD-2015M18 in green identified the breakpoint of the translocation in a 32.1-kb region. Dual color FISH with BAC CTD-2015M18 and RP11-767K3 in red showed a chromosomal inversion involving the *NIPBL* gene. **(B)** UCSC Genome Browser showed the region involved in the translocation on der(15) at 15q26.1. BAC probes are colored in black when mapping on der(15) and in gray when mapping on der(5). BAC probes CTD-2545C19 and RP11-1069H13 in green identified the breakpoint of the translocation in a 17-kb interval region. **(C, D)** FISH results on patient metaphases showed the translocation breakpoints on chromosomes 5 **(C)**, 15 **(D)**, and derivatives. **(E)** Dual color FISH on patient metaphase showed a chromosomal inversion between the region covered by probes CTD-2015M18 and RP11-767K3.

As *NIPBL* was mapped at 5p13.2, we hypothesized that the *de novo* rearrangement was the main cause of the proband’s phenotype and thus refined the translocation breakpoint by targeted FISH experiments. The derivative 5 (der(5)) bkps was mapped at 5p13.2 within the region spanned by probe CTD-2015M18 (Figures 1A, C), whereas the translocation breakpoint on derivative 15 (der(15)) was identified by the 15q26.1 region-specific clones RP11-565O1 and CTD-2545C19 (Figures 1B, D). Based on FISH data, the translocation bkps were mapped within a region of approximately 32.1 kb on der(5) (chr5:33,831,651–33,863,785, hg38) and of approximately 17 kb on der(15) (chr15:90,187,383–90,204,431, hg38). According to the UCSC Genome Browser database, the der(5) breakpoint maps within intron 2 of *ADAMTS12* gene (NM_030955) approximately 3 Mb upstream the *NIPBL* 5′end (Figure 1A), whereas the der(15) breakpoint is localized within intron 1 of the *SEMA4B* gene (NM_020210). FISH experiments with BAC probes covering *NIPBL* did not evidence any gene interruption (Supplementary Figure S2). Further investigations by dual color FISH with probe pairs identified on der(5) at 5p13.2 a paracentric chromosome inversion of at least 4.8 Mb entirely involving *NIPBL* and extending from CTD-2015M18 to RP11-767K3 (chr5:38,416,546–38,604,610 hg38) (Figure 1E).

### 3.5 *NIPBL* expression analysis

**FIGURE 2:**
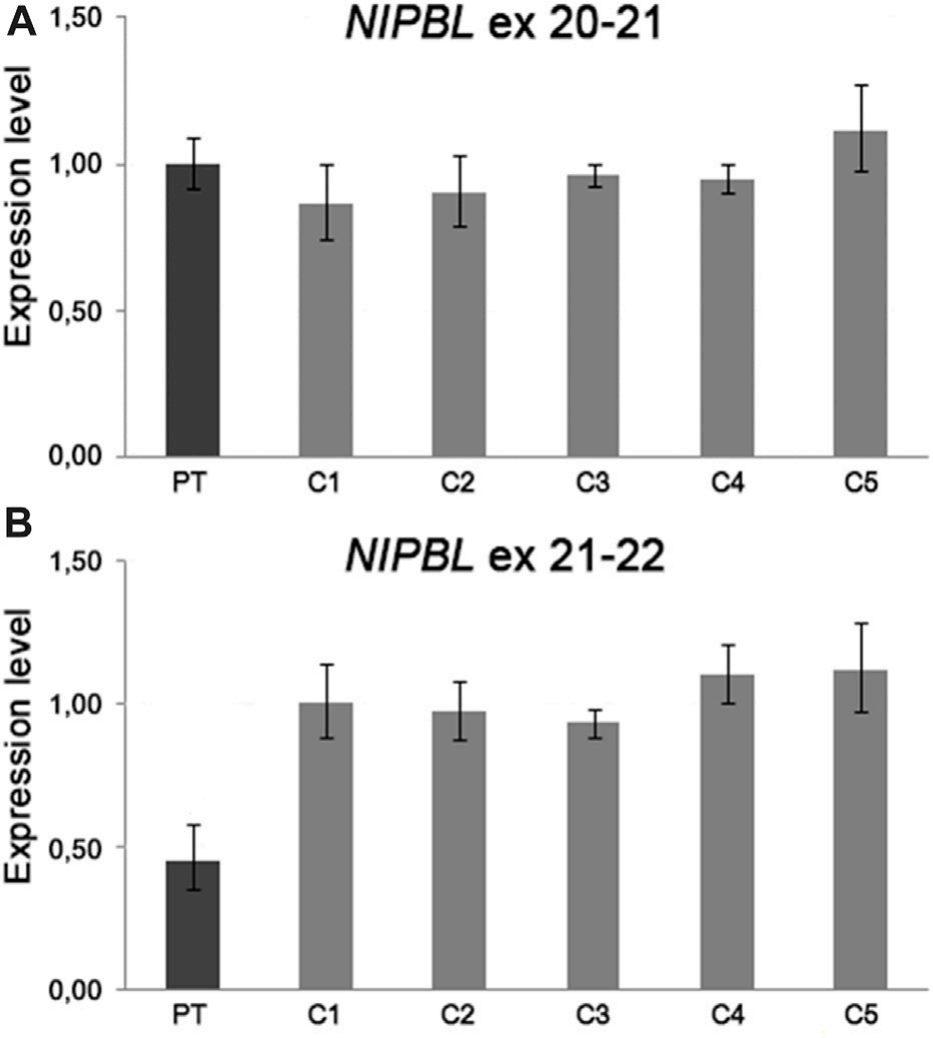
RT-qPCR analysis on patient’s mRNA from peripheral blood showed a *NIPBL* transcript level comparable to five healthy controls using TaqMan probes spanning to the exon junction 20–21 **(A)** and a half amount of mRNA using TaqMan probes spanning from exon junction 21 to 22 **(B)**. The amounts of *NIPBL* mRNAs were calculated in the proband and five healthy controls using the 2^−ΔΔCT^ method, with *GAPDH* and *TBP* as the endogenous normalizing genes. We established the proper range of gene expression in five healthy controls calculating the mean value ±2 standard deviations.

Based on the strong clinical suspicion and to figure out a possible pathogenic alteration of *NIPBL* expression caused by the der(5) translocation breakpoint and paracentric inversion, we performed quantitative gene expression analysis (RT-qPCR) using TaqMan probes. For exon junctions 11–12 and 20–21, *NIPBL* transcript levels were comparable in proband and controls, whereas for exon junctions from 21–22 to 36–37, mRNA levels were approximately 50% decreased in the patient compared to controls (Figure 2). This result pointed to the expression of a truncated *NIPBL* transcript, likely at intron 21, and probably leading to a defective protein, hinting that imbalances of the 5p inverted region might be driven by the CCR. Results of RT-qPCR confirmed the patient’s CDLS1 clinical diagnosis, but the molecular mechanism underlying the translocation event remained unsolved for years.

### 3.6 Nanopore long-read sequencing

**FIGURE 3:**
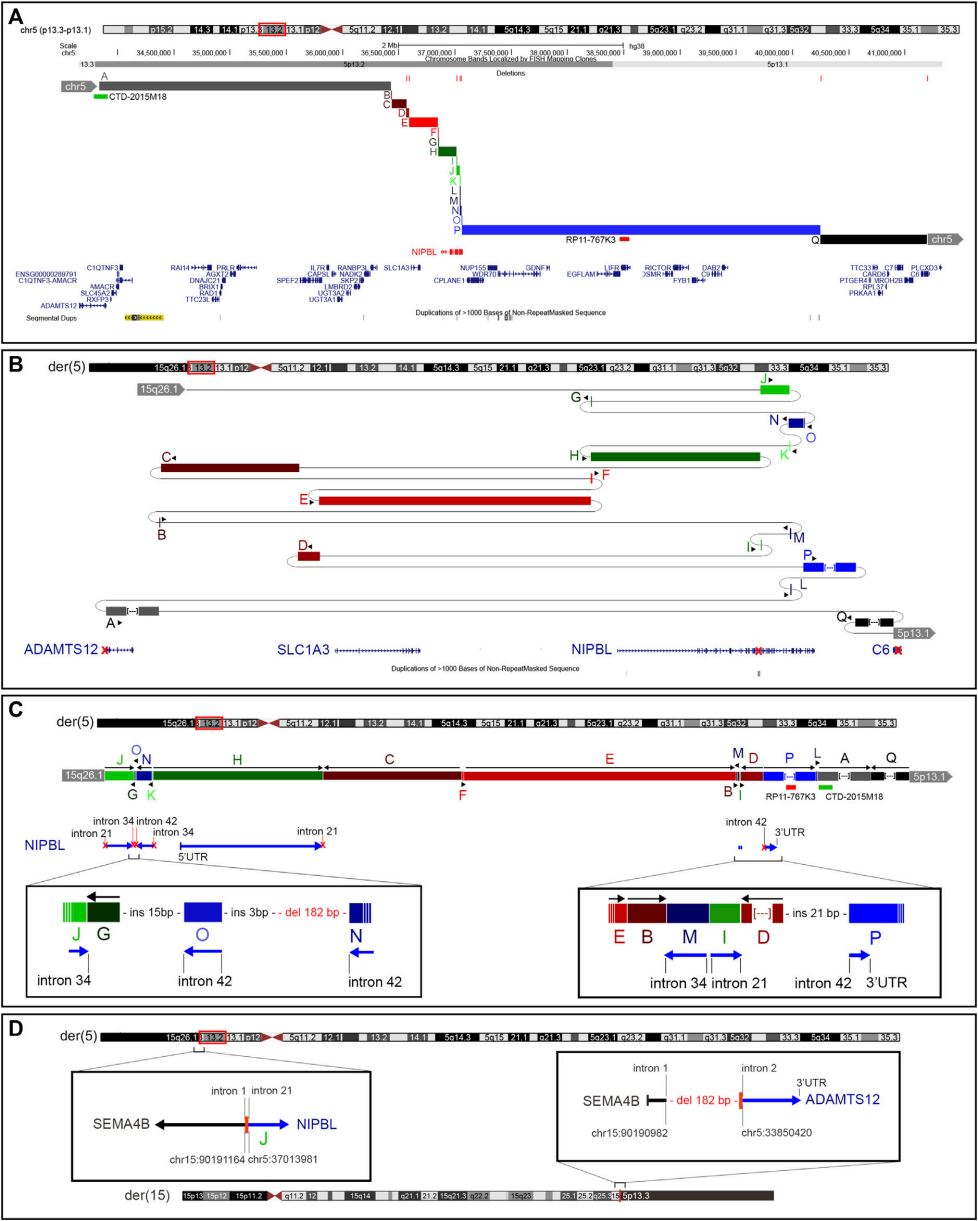
Constitutional chromothripsis event. **(A)** UCSC Genome Browser of the 7.3-Mb region involved in the constitutive complex chromosomal rearrangement. Fragments involved are shown in different colors and letters (A–Q). Deletions occurring at breakpoints are shown in red bars as well as BAC probes used in FISH analysis. **(B)** Enlarged view of the genomic shattering occurred in the *NIPBL* genomic region. Fragments are relocated randomly and some of them are inverted. Fragments A, P, and Q are not in the original size. **(C)** Fragments’ relocation on der(5) with the characterization of the most complex breakpoints involving *NIPBL*. **(D)** Characterization at the nucleotide level of the translocation breakpoints on der(5) and der(15) showing the formation of a fusion gene between *SEMA4B* and *ADAMTS12*.

The LRS approach with ONT produced a total of 28.3 Gb and an average sequencing depth of 8x. Data analysis confirmed the previous breakpoint mapping on derivative chromosomes and allowed to fill the gap in this complex scenario showing the signature of a previous constitutional chromothripsis event on der(5). This led to the shattering at 5p13.2 of a 7.3-Mb region (chr5:33,850,476–41,203,087, hg38) comprising 44 coding genes into 17 distinct fragments (A to Q) with 36 underlying breaks (Figure 3A; Table 2). The fragments vary in length from 48 bp to 3.2 Mb and are relocated in a random order and orientation, with eight of them inverted (Figures 3B, C; Table 2). The analysis at the nucleotide level revealed 13 deletions ranging from 1 bp to 1263 bp occurring at validated breakpoint junctions (Figure 1A; Supplementary Figure S3; Table 3). Most junctions display blunt ends (70%) and some microhomology of 1–5 nucleotides (23%), suggesting the occurrence of non-homologous end joining (NHEJ) or microhomology-mediated break induced repair (MMBIR) as the mechanism for double-stranded DNA breaks (DSBs). Some nucleotide insertions (n = 7) ranging from 1 to 21 bp that do not match with any sequence in the human reference genome have been observed at blunt-end junctions (Table 4).

**TABLE 2:** Fragments from chromothripsis event on der(5).

Fragment	chr	Start^a^	End^a^	Strand	bp	Repeated regions at breakpoint	Interrupted gene
A	chr5	33850476	36438161	+	2587685	SINE MIRb/—	*ADAMTS12 5′UTR to intron 2*
B	chr5	36438162^b^	36438335^b^	+	173	—/SINE MIR	—
C	chr5	36438337	36570907	-	132570	SINE MIR/—	—
D	chr5	36570961	36591614	-	20653	—/—	—
E	chr5	36591628	36851742^b^	+	260114	—/LINE L1PA6	—
F	chr5	36851750	36851817	+	67	LINE L1PA6	—
G	chr5	36851820	36851868	-	48	LINE L1PA6	—
H	chr5	36851872	37013763	+	161891	LINE L1PA6/segmental duplication	*NIPBL 5′UTR to intron 21*
I	chr5	37013772	37013927	+	155	Segmental duplication	*NIPBL intron 21*
J	chr5	37013981	37042438	+	28457	Segmental duplication/SINE AluSg	*NIPBL intron 21 to intron 34*
K	chr5	37042431	37042500	-	69	SINE AluSg	*NIPBL intron 34*
L	chr5	37042516	37042600	+	84	SINE AluSx4	*NIPBL intron 34*
M	chr5	37042644	37042855^b^	-	211	SINE AluSx4/—	*NIPBL intron 34*
N	chr5	37042855	37056005	-	13150	—/LINE L2a	*NIPBL intron 34 to intron 42*
O	chr5	37056188	37056263	-	75	—/—	*NIPBL intron 42*
P	chr5	37056264	40252756	+	3196492	—/—	*NIPBL intron 42 to 3′UTR*
Q	chr5	40252812	41201823	-	949011	LTR/SINE MIR3	*C6 intron 2 to 3′ UTR*

^a^ hg38 genomic coordinates.

^b^ Based on long-read alignment, not validated breakpoints.

AluS, Alu subfamily; LINE, long interspersed nuclear elements; LTR, long terminal repeat elements; MIR, mammalian-wide interspersed repeats; SINE, short interspersed nuclear elements.

**TABLE 3:** Deleted fragments on der(5).

chr	Start^a^	End^a^	bp^b^	Involved gene
chr5	36570908	36570960	53	—
chr5	36591615	36591627	13	—
chr5	37013764	37013771	8	*NIPBL intron 21*
chr5	37013928	37013980	53	*NIPBL intron 21*
chr5	37042501	37042515	15	*NIPBL intron 34*
chr5	37042601	37042643	43	*NIPBL intron 34*
chr5	37056006	37056187	182	*NIPBL intron 42*
chr5	40252757	40252811	55	—
chr5	41201824	41203086	1263	*C6 intron 2*

^a^ hg38 genomic coordinates.

^b^ Only deleted fragments > 3bp are listed.

**TABLE 4:** Breakpoint junction validation on der(5).

Fragment junction^a^	Microhomology (bp)	Blunt ends	Insertion (bp)
**15q26.1–J**	—	+	+ (1)
**J–G**	—	+	—
**G–O**	—	+	+ (15)
**O–N**	—	+	+ (3)
**N–K**	+ (5)	—	—
**K–H**	+ (2)	—	—
**H–C**	+ (1)	—	—
**C–F**	—	+	+ (14)
**F–E**	+ (3)	—	—
**M–I**	—	+	—
**I–D**	—	+	—
**D–P**	—	+	+ (21)
**P–L**	—	+	+ (6)
**L–A**	—	+	—
**A–Q**	—	+	+ (1)
**Q–5p13.1**	—	+	—
**TOTAL**	**4**	**12**	**7**

^a^ Breakpoints adjacent fragment B (E-B and B-M) are not shown as not validated.

Despite the large number of involved coding genes, the catastrophic “all at once” rearrangement on der(5) disrupted the structural integrity of only three genes (i.e., *ADAMTS12*, *NIPBL*, and *C6*) (Figure 3B). Notably, *NIPBL* was the key target gene, with a total of 16 breaks occurring from intron 21 to intron 41 which generated nine fragments relocated in a random way in the 7.3-Mb region (Figure 3C); conversely, a single breakpoint interrupted *ADAMTS12* and *C6* coding sequences both at intron 2.

On der(15), a single breakpoint with a 182-bp deletion was observed (Figure 3D).

## 4 Discussion

The power, sensitivity, and precision of long-read genome technologies are particularly valuable in detecting structural variants at repeated and segmental duplication regions, often found recalcitrant to short-read genome sequencing (Nurk et al., 2022). Although clinically not yet available, LRS proves to be an extremely robust approach for characterizing complex unresolved rearrangements, leading to the identification of variants of larger effect and new models of mutation in Mendelian disease-causing genes (Mastrorosa et al., 2023; Oehler et al., 2023). The commonly used techniques for characterizing chromosomal rearrangements mostly tend to highlight balanced rearrangements without capturing their surroundings and with scarce reconstruction of the generating event.

In this study, the first-tier multilevel approaches of array-CGH, FISH analysis, and RT-qPCR were able to confirm the molecular basis of the clinically diagnosed CDLS patient but were not sufficient to unveil the molecular pathomechanism which remained unsolved for years. The application of long-read sequencing with ONT confirmed the previous first-tier studies and proved decisive: the apparently balanced translocation between the short arm of chromosome 5 and the long arm of chromosome 15 revealed a constitutional chromothripsis event disrupting the *NIPBL* gene, the most frequent cause of CDLS.

Chromothripsis or chromosomal catastrophe has been discovered in human tumors (Stephens et al., 2011), where it is a rather frequent event, whereas its finding at the constitutional level, signaled by complex chromosome rearrangements with more than two breakpoints in patients with congenital disease, is a rarer occurrence with approximately 380 reported cases (Kloosterman et al., 2011; Xing et al., 2022). However, it is increasingly emerging as a mechanism underlying various constitutional pathologies; thanks to the use of second and third next-generation sequencing technologies (Kloosterman et al., 2012; Kurtas et al., 2018; Kurtas et al., 2019; Lei et al., 2020; Mitsuhashi et al., 2020; Plesser Duvdevani et al., 2020; Scharf et al., 2022; Tamura et al., 2023). This study presented an ONT-processed case, where 17 fragments were identified, all originating from chromosome 5, presumably following a local insult to a 7.3-Mb region at bands 5p13.2p13.1, which was accompanied by a single-DSB event on chromosome 15. The outlined rearrangement adheres to all criteria of germline chromothripsis: i) occurrence at the breakpoints of the loss of various genomic regions of different sizes (from a single to >1000 bp) arisen from the shattering process, ii) random insertion of a few bases at the rejoining of various fragments, and iii) the presence of junctions with blunt ends, whereas others exhibit microhomology of sequence (Kloosterman et al., 2011).

Translocations or rearrangements between chromosomes 5 and 15 are rare but described in the literature associated with phenotypes, such as atypical cri-du-chat syndrome (Elmakky et al., 2014), acute lymphoblastic leukemia (Corona-Rivera et al., 2012), or neurodevelopmental disorders (Tamura et al., 2023). The partnership of chromosomes 5 and 15 suggests they may localize to neighboring chromosomal territories in the interphase nucleus. In the study conducted by Mehta et al. (2013), where chromosomal territories were evaluated before and after DNA damage in human fibroblasts, chromosomes 5 and 15 appeared located in non-adjacent positions (periphery and interior, respectively). However, it is conceivable that different repositioning may occur in different cell types (e.g., germ cells), mediating chromosomal exchanges after DSBs.

Although the constitutional rearrangement is confirmed to be *de novo*, it is not currently known whether the chromothripsis event identified in the patient affected the paternal allele, as such events mainly occur in the male germline due to numerous mitotic divisions (Gribble et al., 2005; Kloosterman et al., 2011; Chiang et al., 2012; Pellestor, 2014) or in the early zygote.

It is interesting to note that most breakpoints on der(5), namely, 16, occur at the *NIPBL* locus, suggesting it might be an unstable region prone to rearrangements. Indeed, several breakpoints coincide with repeated sequences, such as SINE (MIR; AluSx4) and LINE (L1PA6), and a segmental duplication resides precisely at intron 21 of the *NIPBL* gene. Reported cases of translocations or CCRs involving *NIPBL* are rare and restricted to a few balanced translocations interrupting the gene (Krantz et al., 2004; Tonkin et al., 2004) or CCRs characterized by array-CGH (Hayashi et al., 2007) or by short-read WGS, with this latter target of a chromothripsis event involving four chromosomes (Plesser Duvdevani et al., 2020). However, considering that approximately 20%–30% of patient with CDLS miss a molecular diagnosis, also due to the high rate of mosaicism (Huisman et al., 2013), it is possible that these complex chromosomal rearrangements might be more frequent than expected, and remain unsolved/undetected by conventional diagnostic procedures.

What emerges from a comparison between short-read and long-read WGS is a different resolution obtained by the two methods (Zhao et al., 2021). In the former, it is not always possible to obtain all breakpoints, map them precisely, and validate with other methods, thus missing the reconstruction of involved whole genomic regions. Conversely, the LRS approach may unveil a catastrophic event with a high-resolution identification of numerous genomic fragments (Lei et al., 2020).

Even in the present case, the LRS approach allowed for a high resolution of the rearrangement, identifying very small fragments (48 bp) and leading to the validation of all breakpoints through Sanger sequencing. The analysis was crucial as highlighted for additional breaks and fragments in four breakpoints’ validations (data not shown).

Although 44 genes were mapped to the 5p13.2p13.1 region where the catastrophic event had occurred, the majority of DSBs hit *NIPBL* with only *ADAMTS12* and *C6* both interrupted at intron 2. The repositioning of various fragments did not generate any fusion gene on der(5). In contrast, the juxtaposition at der(15) between the short arm of chromosome 5 and the long arm of chromosome 15 led to the formation of a fusion gene between *SEMA4B* (5′UTR—intron 1) and *ADAMTS12* (intron2—3′UTR). Considering that exon 1 of the *SEMA4B* gene is part of the UTR and exon 3 of the *ADAMTS12* gene is coding but lacks the start codon (ATG), it may be assumed that no transcription occurs and no alternative start codon is recognized at der(15). Thus, the possibility that the rearranged der(15) may contribute to the clinical manifestations presented by the proband appears remote.

## 5 Conclusion

In conclusion, we reported a peculiar case of a patient with a diagnosis of CDLS1 caused by a constitutive chromothripsis event that involved chromosomes 5p and 15q. Using a multilevel genomic approach, we were able to confirm the clinical diagnosis by finding a *NIPBL* disrupted transcript, but the complex and complete scenario and the pathomechanism causing this alteration were reconstructed using long-read sequencing. Our findings highlight that apparently balanced translocation may sometimes be more complex and the LRS approach deserves to be considered when conventional procedures fail to detect genomic alteration in patients with a clear clinical suspicion, who are suspected to have complex chromosomal rearrangements.

## Data Availability

The data presented in the study are deposited in the Harvard Database repository (https://dataverse.harvard.edu/), accession number/link https://doi.org/10.7910/DVN/HXAGSD.
