# Supplementary material for: Long-read sequencing reveals chromothripsis in a molecularly unsolved case of Cornelia de Lange syndrome
Source: Front Genet. 2024 Mar 13;15:1358334. doi: 10.3389/fgene.2024.1358334 (PMC10965544; doi:10.3389/fgene.2024.1358334)
Supplement: Supplementary file 1 [file DataSheet1.docx]

Supplementary Material

# Supplementary Figures and Tables

## Supplementary Figures


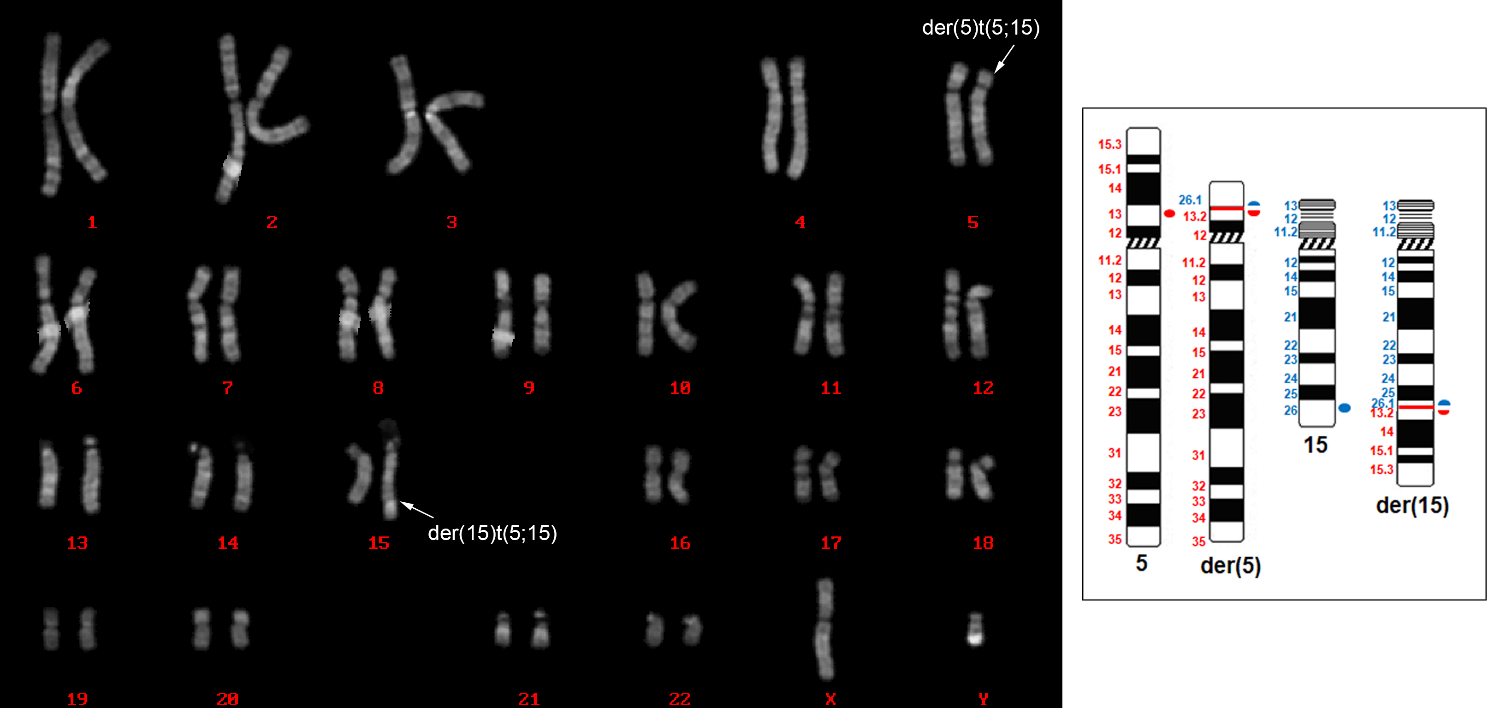


**FIGURE S1.** Patient’s karyotype showing the translocation between chromosomes 5 and 15. Ideogram of the involved chromosomes is shown on the right.


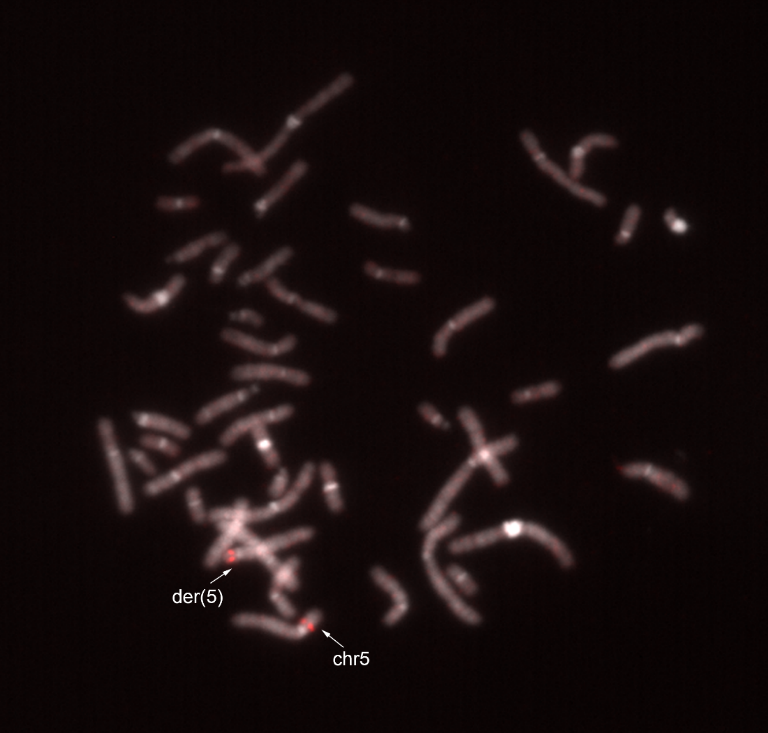


**FIGURE S2.** FISH analysis performed using BAC clone CTD-2047K3 covering *NIPBL* gene revealed comparable signals between chromosome 5 and derivative 5 without breakpoint identification and showing no gene interruption.


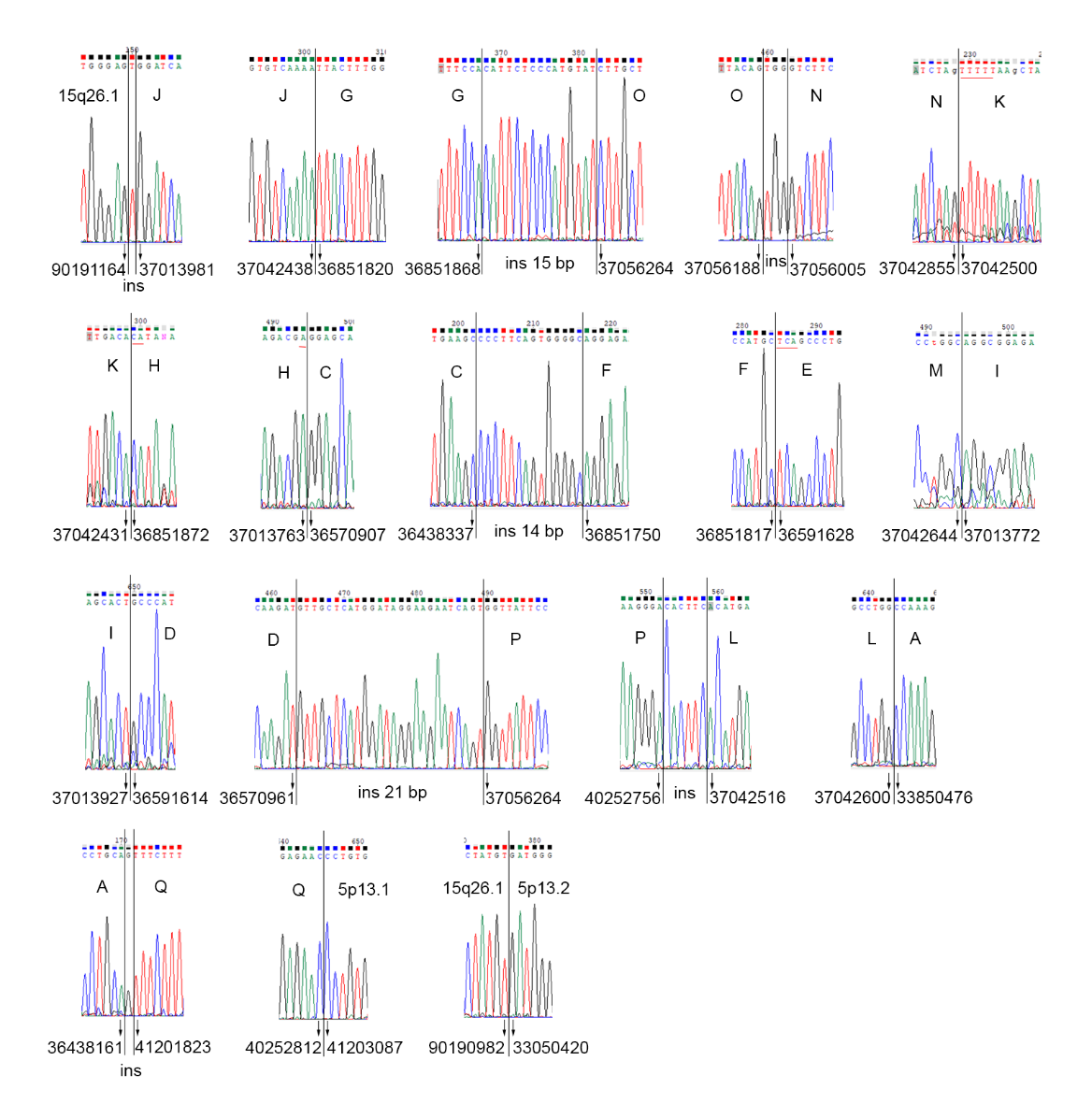


**FIGURE S3.** Sanger validation of the breakpoints generated by the catastrophic event on chromosome der(5) and der(15). Hg38 cohordinates are shown in the breakpoint junction for each fragment (A to Q). Validation of breakpoint covering fragment B was not performed. Bases underlined in red represent the microhomology regions.

## Supplementary Table

| TABLE S1 Primers used for breakpoints validation | |  |
| --- | --- | --- |
| **Primer ID** | **Seq 5' -> 3'** | **Estimated product size (bp)** |
| BKPder5_F | CCTTCTGGGTACTCCCTCAA | 384 |
| BKPder5_R | TAAGCTCTCCctccctctcc |  |
| BKP1 F | CCAAGTGGTGGGAATGTTTT | 709 |
| BKP1 R | CAGTCAAGCAGTGAGGGACA |  |
| BKP2 F | catgctcagccTAGGACTTCTT | 227 |
| BKP2 R | TGGGGTATATTAGCTAGGCCTCt |  |
| BKP3 F | agtgagcttgagcccctttt | 900 |
| BKP3 R | GGTCACAGCTGACCACATGA |  |
| BKP4 F | TGACTGAAAAGATTCCATCAAAA | 205 |
| BKP4 R | GCATCCATGGGATTGCTG |  |
| BKP5 F | CTAGGCTTCAAGGTGAGATGC | 1304 |
| BKP5 R | TCAGTGACACTTAGAGAATGATTTTTG |  |
| BKP6 F | CTCAAGGCAAACTATTCAAAAGC | 600 |
| BKP6 R | AAAAACACTCACTCCTACCATCTACC |  |
| BKP7 F | AAGATAAATTGTTTCTTTTGATTCCTC | 651 |
| BKP7 R | TTATAGAACTCAGGAACTCTGACCAA |  |
| BKP8 F | GCATGCATGAGAATGGTGAC | 650 |
| BKP8 R | AATGGAATTTTCCCTCAATGG |  |
| BKP9 F | agcctcaCTTCCTCCAGGTT | 1376 |
| BKP9 R | CTGGAGGTTGTGCCTATGGT |  |
| BKPder15_F | GTTGGTTTCCTGAGGTGGAG | 400 |
| BKPder15_R | TCTCACATGCCTCTGGGTTT |  |
